# Stiffness in der Kompressionstherapie

**DOI:** 10.1007/s00105-025-05546-5

**Published:** 2025-08-18

**Authors:** Dennis Braß, Markus Stücker

**Affiliations:** Klinik für Dermatologie, Venerologie und Allergologie, Venenzentrum der dermatologischen und gefäßchirurgischen Kliniken, Katholisches Klinikum Bochum gGmbH, Gudrunstr. 56, 47791 Bochum, Deutschland

**Keywords:** Kompressionsstrumpf, Kompressionsdruck, Chronische venöse Insuffizienz, Massageeffekt, Venöse Hämodynamik, Compression stocking, Compression pressure, Chronic venous insufficiency, Massage effect, Venous hemodynamics

## Abstract

**Hintergrund:**

Die therapeutischen Effekte von Kompressionsstrümpfen werden neben dem Druck, gekennzeichnet über die Kompressionsklassen I–IV, maßgeblich auch von den elastischen Eigenschaften des Materials in Bewegung bestimmt. Diese dynamischen Eigenschaften von Kompressionsstrümpfen wirken sich auf den in Bewegung stattfindenden Massageeffekt an der unteren Extremität aus und lassen sich mithilfe der Stiffness des Kompressionsmaterials beurteilen. Die Stiffness beschreibt hierbei die Zunahme des Kompressionsdrucks unter Belastung durch die Umfangszunahme des Unterschenkels und lässt sich mithilfe verschiedener Methoden sowohl in vitro als auch in vivo bestimmen.

**Ziel der Arbeit:**

Ziel unserer Übersichtsarbeit ist es, die Thematik der Stiffness von Kompressionsmitteln in ihren verschiedenen Aspekten zu beleuchten, um das Bewusstsein für diese therapeutisch relevante Einflussgröße zu schärfen.

**Material und Methoden:**

Es erfolgte von Juli 2024 bis September 2024 eine webbasierte Literaturrecherche zu Arbeiten rund um das Thema Stiffness, Kompressionstherapie und Kompressionsmaterialien in den Datenbanken von PubMed sowie in online zugänglichen Fachzeitschriften und gedruckter Literatur.

**Ergebnisse und Diskussion:**

Die Stiffness stellt einen gesonderten Prädikator für die Effektivität einer Kompressionstherapie dar und kann im klinischen Alltag gezielt eingesetzt werden, um die optimale Kompressionsmittelversorgung für die unterschiedlichen Indikationen und Patienten auszuwählen. Wünschenswert wäre in Zukunft ein Konsensus zur Ermittlung der Stiffness von medizinischer Kompressionsversorgung. Dies würde es den Herstellern von Kompressionsmitteln ermöglichen, Stiffness als Produktinformation zu etablieren, um die aktuell bestehende Informationslücke auf dem Markt zu schließen.

## Abstract (en)

**Background:**

The therapeutic effects of medical compression devices is determined not only by the resting pressure exerted at the ankle, which determines the compression classes I–IV, but also to a large extent by the elastic properties of the material during movement. These dynamic properties of compression stockings affect the massage effect on the lower extremity during movement and can be assessed using the stiffness of the compression material. Stiffness describes the increase in compression pressure under load due to the increase in circumference of the lower leg and can be determined using various methods both in vitro and in vivo.

**Aim:**

The aim of our review is to shed light on the various aspects of the stiffness of compression devices in order to raise awareness of this therapeutically relevant influencing factor.

**Materials and methods:**

From July 2024 to September 2024, a web-based literature search was conducted on the topic of stiffness, compression therapy and compression materials in PubMed, as well as in online journals and printed literature.

**Results and conclusion:**

Stiffness is a separate predictor for the effectiveness of compression therapy and can be used specifically in everyday clinical practice to select the optimum compression garment for different indications and patients. In the future, it would be desirable for manufacturers of compression products to clearly define stiffness in the product information in order to close the current information gap in the market.

Die medizinische Kompressionstherapie ist ein Pfeiler in der konservativen Therapie von lymphologisch-phlebologischen Krankheitsbildern. Sie bietet eine Vielzahl an unterschiedlichen Modalitäten zur Versorgung der unterschiedlichen Patientengruppen und Krankheitsbilder. Neben dem Kompressionsdruck spielen insbesondere die elastischen Eigenschaften des Materials einen entscheidenden Faktor in der Therapieeffektivität. Eine Quantifizierung dieser Eigenschaften kann über die Stiffness der Kompressionsmaterialien erfolgen.

## Kompressionstherapie

Die medizinische Kompressionstherapie stellt eine tragende Therapiesäule in der konservativen Behandlung venöser und lymphatischer Erkrankungen dar, so unter anderem in der Therapie der chronischen venösen Insuffizienz inklusive venös bedingter Unterschenkelulzera, der akuten tiefen Beinvenenthrombose oder des akuten und chronischen Lymphödems [1, 2].

Effekte der Kompressionstherapie umfassen – unter anderem über eine Reduktion des Gefäßdurchmessers – die Verbesserung der Strömungsverhältnisse innerhalb der venösen Gefäße, die Reduktion des venösen Refluxes, damit einhergehend eine Reduktion des ambulatorischen venösen Hochdrucks, eine Verbesserung der Filtrations‑/Resorptionsverhältnisse innerhalb der Kapillarstrombahn und hierüber eine Reduktion auftretender Gewebsödeme sowie eine Reduktion des Risikos für das Auftreten von Ulzera [3, 4].

Weiterhin schaffen sie subjektive Linderungen bei Symptomen wie Schweregefühl der Beine, Schmerzen oder Juckreiz [2, 3]. Klassifiziert werden die verschiedenen medizinischen Kompressionsstrümpfe derzeit nach RAL-GZ 387 (Reichs-Ausschuss für Lieferbedingungen, Deutsches Institut für Gütesicherung und Kennzeichnung e. V.). Es erfolgt hiernach eine Einteilung in die Kompressionsklassen I–IV nach dem vorliegendem Ruhedruck, also anhand des in vitro bestimmten Anpressdrucks der verschiedenen Strümpfe im Bereich der Fußfessel (B-Level) [3, 5]. Anhand der absoluten Drücke in Millimeter pro Quecksilbersäule (mm Hg) ergeben sich daraus die Kompressionsklassen:

I: 15–21 mm Hg; II: 23–32 mm Hg; III: 34–46 mm Hg; IV: ab 49 mm Hg.

Für die hämodynamisch positiven Aspekte von Kompressionsstrümpfen sind v. a. zwei Eigenschaften verantwortlich. Neben dem Kompressionsdruck spielen die dynamischen Eigenschaften des Materials eine entscheidende Rolle, da sie maßgeblich den in Bewegung auftretenden Massageeffekt an der unteren Extremität mitbedingen [6–8]. Diese dynamischen Eigenschaften werden v. a. durch die Stiffness des Materials bestimmt, welche ihrerseits die Druckzunahme zwischen Kompressionsstrumpf und Unterschenkel unter Zunahme des Unterschenkelumfangs beschreibt [9–16].

Zur In-vitro- und In-vivo-Messung und Berechnung von Parametern wie Stiffness, Ruhedruck oder Arbeitsdruck wurden spezifische Messpunkte festgelegt, welche Tab. 1; [17] zu entnehmen sind.

**Tab. 1:** Messpunkte der unteren Extremität

Messpunkt	Lokalisation des Messpunktes
a	Fußsohle an der Ferse
A	Vorfuß am Ansatz der Zehen
B	Knöchel an der Stelle des geringsten Umfangs
B1	Punkt, an dem die Achillessehne in die Wadenmuskulatur übergeht
C	Wade an ihrem maximalen Umfang
D	Höhe der Tuberositas tibiae
E	Mitte der Kniescheibe und unter der Kniekehle hindurch
F	Zwischen K und E
G	5 cm unterhalb von K bei aufrechter Haltung des Patienten
H	Gesäß an der breitesten Stelle des Trochanters
K	Mittelpunkt des Schritts
K1	Höhe der Schambeinfuge
K2	Höhe der Glutealfalte
T	Natürliche Taillenlinie
X	Mitte des Fußes
Y	Fußspann
Z	Zehenspitze

Quelle: Partsch H, Rabe E, Stemmer R. Kompressionstherapie der Extremitäten. Editions Phlébologiques Françaises © 1999; 1. Auflage. Kap. 5 Kompression: 73–74 [17]

## Definition von Stiffness

Die Stiffness beschreibt die Festigkeit eines Kompressionsmittels, in anderen Worten den materialbedingten Widerstand gegen Verformung und Dehnung bei applizierter Kraft [2, 6]. Sie wird in Millimeter pro Quecksilbersäule (mm Hg) oder Hektopascal (hPa) angegeben [10] und ist definiert als Zunahme des Kompressionsdrucks pro Zunahme des Beinumfanges um 1 cm am entsprechenden Messpunkt [2, 6, 9–12, 14, 15, 18, 19].

Im Kontext der Kompressionsbestrumpfung stellt die Stiffness eine relevante Produkteigenschaft insbesondere im Stand und in Bewegung dar [10, 13–15, 18–22].

Während der Muskelsystole kommt es zu einer Zunahme des Unterschenkelumfangs, was bei anliegendem Kompressionsmittel zu einer Dehnung des Materials führt. Der zyklische Wechsel aus Dehnung und Entspannung des Kompressionsmaterials, also der Wechsel aus Ruhedruck und Arbeitsdruck, bewirkt am Bein einen „Massageeffekt“ mit hämodynamisch positiven Einflüssen auf das venöse Gefäßsystem [3, 4, 6–8, 10, 12, 16, 18, 20]. Inwieweit die verwendeten Fasern im Kompressionsmittel eine Dehnung erlauben bzw. unter welcher Druckzunahme diese Dehnung erfolgt, wird durch die Stiffness beschrieben.

## Quantifizierung der Stiffness

Die Stiffness verschiedener Kompressionsmaterialien kann mit dem statischen Stiffness-Index (SSI) und dem dynamischen Stiffness-Index (DSI) sowohl in vitro als auch in vivo bestimmt werden.

### Statischer Stiffness-Index (SSI)

Der statische Stiffness-Index ist eine in vivo am Menschenbein mit angelegtem Kompressionsmittel berechnete Messgröße und definiert sich als Differenz aus Ruhedruck im Liegen und dem Anpressdruck im Stehen [2, 5, 6, 8–10, 13, 14, 18, 21, 23–25].

Da die zur Berechnung der Stiffness definitionsgemäße Zunahme des Unterschenkelumfanges von 1 cm näherungsweise am ehesten am B1-Punkt erfolgt, werden die dort erhobenen Druckmessungen von Ruhedruck und Anpressdruck zur Berechnung des SSI herangezogen.

Der statische Stiffness-Index gibt Aufschluss über die Druckzunahme verschiedener Kompressionsmittel unter realen Bedingungen und stellte damit eine für Patient*innen klinisch relevante Messgröße dar. Bei einem SSI von < 10 mm Hg spricht man von elastischen Materialien, ein SSI von > 10 mm Hg definiert unelastische Kompressionsmittel [14, 26, 27].

#### Merke.

Statischer Stiffness-Index (SSI): Berechnung als Differenz aus dem Anpressdruck im Stand und dem Ruhedruck im Liegen.

### Dynamischer Stiffness-Index (DSI)

Der dynamische Stiffness-Index ist eine berechnete Größe, welche die Druckzunahme unterhalb eines Kompressionsmittels in Bewegung beschreibt. Dieser unter Bewegung auftretende Kompressionsdruck wird als Arbeitsdruck bezeichnet. Definiert ist der dynamische Stiffness Index als Zunahme des Drucks, wenn die Veränderung des Unterschenkelumfangs 1 cm bei einer Frequenz von 1 Hertz (1 Hertz = 1 Gangzyklus pro Sekunde) beträgt [10, 21].

Der DSI kann verwendet werden, um die Eigenschaften eines Kompressionsstrumpfes in Bewegung zu beschreiben, genauer gesagt die durch Zu- und Abnahme des Unterschenkelumfanges/-radius in Muskelsystole und -diastole erfolgten Druckänderungen am Bein [4].

In den DSI fließt die Hysteresis eines Kompressionsmaterials ein, also der Verlust an wiedergewonnener linearer Länge des elastischen Materials nach repetitiver Dehnung und Entspannung der Fasern [11].

Bestimmen lässt sich der DSI in vitro unter Verwendung eines „dynamic leg-segment models“ [10, 12, 20]. In-vivo-Messungen der dynamischen Stiffness erfolgten durch verschieden definierte, aktive Bewegungen am Patientenbein. So wurden die Maximaldifferenz des Kompressionsdruckes während Extension und Flexion des Sprunggelenks [1, 2, 8, 13, 19] als auch die Differenz des Maximal- und Minimaldrucks während des Zehenspitzenganges [23] bzw. -standes [6] zur Berechnung des DSI herangezogen. Eine andere Berechnung des DSI erfolgte als Bestimmung der Differenz zwischen dem Kompressionsdruck im entspannten Stand und Zehenspitzenstand [5].

#### Merke.

Dynamischer Stiffness-Index (DSI): Zunahme des Kompressionsdrucks bei Zunahme des Unterschenkelumfangs um 1 cm bei einer Frequenz von 1 Hertz; 1 Hertz = 1 Ganzzyklus pro Sekunde.

## Statischer Stiffness-Index (SSI) und dynamischer Stiffness-Index (DSI)

### Welcher Parameter eignet sich in der täglichen Praxis?

In der klinischen Praxis ist sowohl die Bestimmung des SSI (in vivo/in vitro), als auch die Bestimmung des DSI (in vivo/in vitro) möglich. Der SSI stellt hierbei eine einfach zu bestimmende Messgröße dar, während der DSI einen beachtlichen Mehraufwand erfordert.

Zwar zeigten sich in der Bestimmung des DSI höhere Stiffness-Werte als beim SSI, gleichzeitig konnte jedoch eine signifikante Korrelation zwischen den Druckwerten der statischen und dynamischen Stiffness gezeigt werden [6, 8–10, 14], weshalb die Bestimmung des SSI für die Entscheidungsfindung des richtigen Kompressionsmittels in der klinischen Praxis als ausreichend anzusehen ist [10].

### Messpunkte

Die verschiedenen Messpunkte zur Bestimmung der Umfangsmaße der unteren Extremität, welche ebenfalls als Lokalisationen für Druckmessungen unter Kompressionsmitteln in vivo fungieren, finden sich in Tab. 1. Im Rahmen von in vitro- und in vivo-Messungen werden unterschiedliche Messpunkte ausgewählt bzw. fokussiert.

Während Hersteller von Kompressionsartikeln sich bei in vitro-Messungen zum Kompressionsdruck v. a. am B‑Punkt orientieren, also am Bereich des Knöchels mit dem geringsten Durchmesser, erfolgen in vivo-Messungen unter Hinzunahme und besonderer Gewichtung des B1-Punktes [6, 9, 10, 12, 14, 15, 18–20, 22, 28], also dem Übergang der Achillessehen in die Muskelbäuche der Wadenmuskulatur (vgl. Tab. 1).

Entscheidend für die geänderte Gewichtung der Messlokalisation bei in vivo-Messungen im Vergleich zu in vitro-Messungen ist die anatomisch herausfordernde Lage des B‑Punktes. Aufgrund von Knochenvorsprüngen und Sehnen sowie durch die Fossa retromalleolaris, welche durch ihre konkave Form einen Hohlraum darstellt, bestehen am menschlichen Bein kein einheitlicher Radius sowie keine einheitlichen Spannungsverhältnisse mit gleichmäßigem Anliegen von Kompressionsstrümpfen [18]. Diese Tatsache erschwert die Einhaltung der allgemeinen Empfehlungen zu Messtechniken und Messsensoren (vgl. Tab. 2 und 3; [18]). Zudem stellt der Übergang der Achillessehne in die Wadenmuskulatur den anatomischen Bereich mit der größten Umfangsänderung in Bewegung dar [6, 9, 10, 12, 18–20, 28]. In diesem Bereich kann vom größten Einfluss der Stiffness auf den unter Bewegung stattfindenden Massageeffekt der unteren Extremität ausgegangen werden [6, 9, 18, 20].

**Tab. 2:** Empfohlene Messtechniken zur Bestimmung des Kompressionsdrucks der unteren Extremität

Empfohlene Messtechniken
Dünner und flexibler Sensor
Sensormessflächen sollten an ihre vorgesehenen Messgebiete angepasst sein, am Bein z. B. > 5 cm^2^
Sensoren sollten keine Hautirritationen verursachen oder an Messgenauigkeit verlieren
Sensoren sollten kontinuierliche Druckmessung bei aktiver oder passiver Bewegung erlauben
Einfache Kalibrierung der Sensoren
Druckmessungen mit multiplen Messsensoren an verschiedenen Lokalisationen zur gleichen Zeit sind Einzelsensoren vorzuziehen

Quelle: „Measurement of lower leg compression in vivo: recommendations for the performance of measurements of interface pressure and stiffness: consensus statement“ [18]

**Tab. 3:** Eigenschaften des „idealen Sensors“ zur Druckmessung

Eigenschaften des „idealen Sensors“
Größe: unempfindlich gegenüber punktuellen Druckspitzen
Flexibilität: unempfindlich gegen Biegung, aber nicht dehnbar
Haltbarkeit
Reliabilität
Überlastungstoleranz
Elektronische Einfachheit
Auflösung (Zeit < 0,1 s, Amplitude < 0,1 mm Hg)
Betriebsbereich im Einklang mit biologischen Parametern
Geringe Hysterese
Geringe Kosten
Variable Sensorgrößen
Dünn
Hohe Genauigkeit
Hohe Messrate für Messungen in Bewegung
Lineare Reaktion auf den ausgeübten Druck
Kontinuierliche Messungen
Geringe Verformung
Unempfindlich gegen Temperatur- und Feuchtigkeitsschwankungen

Quelle: „Measurement of lower leg compression in vivo: recommendations for the performance of measurements of interface pressure and stiffness: consensus statement“ [18].

Relevant wird dieser Unterschied zwischen den Messpunkten bei Betrachtung des Laplace-Gesetzes [10, 29].

#### Merke.

P = S/r

P = Druck; S = Streckung des Materials; r = Radius des Unterschenkels

Quelle: Moderne Kompressionstherapie. Ein praktischer Leitfaden. Viavital Verlag GmbH 2013; 1. Auflage. Kap. 1; S. 15 [29]

Dieses beschreibt im Kontext der Kompressionstherapie die Abhängigkeit des ausgeübten Drucks eines Kompressionsstrumpfes von der Spannung des Materials und dem Radius des Unterschenkels. Mit Zunahme des Unterschenkelumfangs, z. B. durch Muskelkontraktion oder Ödeme, steigt bei zunehmenden Spannungsverhältnissen des Kompressionsmittels der ausgeübte Kompressionsdruck [9, 11, 26]. Visualisiert werden kann dieser Zusammenhang in Form von Druck-Umfangs-Kurven [11] und Kraft-Dehnungs-Kurven [12], welche die Zunahme des Kompressionsdrucks bei Zunahme des Unterschenkelumfangs zeigen. Eine starke Steigung, also eine hohe Druckzunahme bei geringer Umfangsvermehrung des Unterschenkels bzw. Dehnung des Kompressionsmittels ist gleichbedeutend mit einer hohen Stiffness des Materials [4, 11, 12, 14].

### Messmethoden

#### In vitro-Messungen

In vitro-Messungen zur Stiffness erfolgen unter kontrollierten Bedingungen im Labor und können sowohl durch Hersteller von Kompressionsartikeln als auch im Rahmen von Forschungsarbeiten durchgeführt werden.

Die Beurteilung der Stiffness in vitro orientiert sich an der festgelegten Definition, d. h. über die Zunahme des Kompressionsdrucks pro Zunahme des Beinumfanges um 1 cm am entsprechenden Messpunkt [2, 6, 9–12, 14, 15, 18, 19].

Bis dato wurde kein einheitliches Messsystem zur in vitro-Bestimmung der Stiffness etabliert, sodass weltweit unterschiedlichste Verfahren zur Anwendung kommen [18, 22].

Einigen dieser Methoden ist das komplexes Aufgebot an Messinstrumenten gemeinsam, wodurch sie im klinischen Alltag nicht praktikabel sind. Hierunter fallen z. B. Messungen mittels HOSY (Forschungsinstitut Hohenstein) in Deutschland oder HATRA (Segar Design) in Großbritannien [6, 22].

#### In vivo-Messungen

In vivo-Messungen erfolgen unter realen Bedingungen am Probandenbein und stellen hierdurch eine Abbildung der Stiffness unter alltagsnahen Bedingungen dar, da individuelle Patientenfaktoren, wie die natürlichen anatomischen Gegebenheiten und Strukturen der unteren Extremität, Muskelaktivitäten sowie Umweltfaktoren, wie z. B. die Umgebungstemperatur, in die Messungen einfließen [4, 15, 18, 29].

Da eine Bestimmung der Druckänderung pro cm Umfangsänderung in vivo unpraktikabel ist, verwendet man hier den statischen Stiffness-Index zur Abbildung der elastischen Eigenschaften eines Kompressionsstrumpfes [6].

### Messtechniken zur in vitro- und in vivo-Bestimmung der Stiffness

#### Medical Stocking-Tester (MST, Salzmann Medico, St. Gallen, Switzerland)

Der Medical Stocking-Tester (MST, Salzmann Medico, St. Gallen, Switzerland) kann verwendet werden zur Bestimmung von Kompressionsdruck und Stiffness [9].

Dieses System erlaubt sowohl in vitro- wie auch in vivo-Messungen und besteht aus einem flachen, luftgefüllten Schlauch, welcher zur Messung des Interface-Pressures (= Kompressionsdruck) zwischen einem Kompressionsstrumpf und einem Holzbeinmodell (in vitro) oder einem Probandenbein (in vivo) angelegt wird. Insgesamt 4 elektrische Kontakte registrierten den erzeugten Kompressionsdruck an vier verschiedenen Messpunkten: B, B1, C, D (vgl. Tab. 1; [2, 9]). Zur Bestimmung der Stiffness-Indizes in vitro wird ein transversaler Streifen des Kompressionsstrumpfes auf Höhe des B1-Messpunktes herausgeschnitten und in ein Zwick-Dynamometer (Zwick-Roell, Ulm, Deutschland) eingespannt. Anschließend erfolgen 6 Zyklen, in denen der Strumpfausschnitt überstreckt wird, wobei im letzten Durchgang die Beziehung aus Dehnungskraft (in Newton [N]) und Dehnung des Stoffes (in cm) zur Berechnung des Kompressionsdrucks und der Stiffness herangezogen wird.

In vivo erfolgen nach Anlegen des MST und Kompressionsstrumpfes Druckmessungen in liegender und stehender Position an den 4 zuvor genannten Messpunkten [9].

Zur Bestimmung des statischen Stiffness-Index erfolgte die Bestimmung der Differenz aus Anpressdruckdruck (Stand) und Ruhedruck (Liegen) am B1-Punkt [9].

#### Instron-Tester (Instron International Ltd., Edegem, Belgium)

Der Instron Tester (Instron International Ltd., Edegem, Belgium) ist eine in vitro-Testmethode zur Bestimmung von Kompressionsdruck und Stiffness und die von der CEN (Comité Européen de Normalisation) empfohlene Testmethode zur Bestimmung von Kompressionseigenschaften bei Kompressionsstrümpfen [11].

Der Instron-Tester ist ein modifiziertes Tensiometer, in welches ein zu untersuchender Kompressionsstrumpf zwischen 2 T-Stäben eingespannt wird, um den dazwischenliegenden Kompressionsdruck zur bestimmen. Hierzu wird der eingespannte Strumpf an zuvor definierten Messpunkten markiert, anschließend die dort vorliegende Zirkumferenz des Strumpfes gemessen und ein Zyklus aus insgesamt 6 Durchgängen durchgeführt, in denen der Kompressionsstrumpf auf seine maximale Zirkumferenz gedehnt wird. Die maximal benötigte Kraft im sechsten Durchgang wird verwendet, um den Kompressionsdruck zu berechnen [11].

#### Institut de Textile France-Methode und „dynamic leg segment model“

Zwei weitere Systeme zur in vitro-Messung der statischen und dynamischen Stiffness stellen die Institut de Textile France-Methode und das „dynamic leg segment model“ dar.

Mittels Institut de Textile France-Methode erfolgt die Bestimmung der statischen Stiffness analog zum zuvor genannten Instron-Tester [10].

Zur Bestimmung des dynamischen Stiffness-Index beschrieben van der Wegen-Franken et al. erstmals das „dynamic leg segment model“ [10, 12, 20]. Dieses besteht aus einem künstlichen Beinsegment, aufgebaut aus einer luftgefüllten Trommel mit darüber liegender Gummihaut, welches in seinem Durchmesser dem Durchmesser eines menschlichen Beines am B1-Punkt entspricht. Mittels eines Luftdruckgenerators kommt es zur Nachbildung des natürlichen Gangzyklus am menschlichen Bein mit zyklischen Druckänderungen und Umfangsänderungen des artifiziellen Beinmodells durch Zugabe und Wegnahme von Pressluft. Die dabei entstehenden Druckmesswerte werden digital aufgezeichnet und zur Berechnung der dynamischen Stiffness herangezogen [10, 12, 20].

#### Pneumatische Messonden: Picopress, Kikuhime, AMI-3037 („mannequin leg model“)

Eine weitere Messmodalität zur Bestimmung von Kompressionsdruck und Stiffness stellt die Gruppe der pneumatischen Messsonden dar. Das CE-zertifizierte Picopress-System (Microlab, Padova, Italy) [1, 2, 6, 7, 13, 23] nutzt eine 5 cm messende und < 1 mm dicke Sonde, welche unter Inflation von 2 ml Luft den Druck zwischen Kompressionsmittel und Bein unter in vitro- oder in vivo-Bedingungen misst. Aus den so erhobenen Daten wird eine Basis zur Berechnung der Stiffness-Indizes generiert [13].

Eine weitere, nach gleichem Prinzip funktionierende Messsonde stellt das Kikuhime-System (TT Medi Trade, Soleddet 15, DK 4180 Soro) dar, welches ein 30 × 38 mm durchmessendes und 3 mm dickes Luftkissen nutzt und ebenfalls für in vitro- und in vivo-Messungen herangezogen werden kann [14, 29]. Aufgrund einer fehlenden CE-Zertifizierung wird dieses System derzeit nicht in der Europäischen Union und dem Vereinigten Königreich vermarktet.

Das von Hirai et al. erstmalig beschriebene „mannequin leg model“ [15, 22] beschreibt eine Methode zur in vitro-Bestimmung von Kompressionsdruck und Stiffness am B1-Punkt unter Verwendung einer derzeit nicht CE-zertifizierten, somit nicht in der europäischen Union oder dem Vereinigten Königreich erhältlichen, pneumatischen Messsonde (Model AMI-3037, AMI Co, Japan). Bei diesem Testverfahren wird ein künstliches Beinmodell in seiner Gesamtlänge längs gespalten und mit einer Hebelmaschine kombiniert. Durch Nutzung der Hebelmaschine lassen sich die beiden Unterschenkelhälften um 5 mm voneinander entfernen, sodass der Umfang des Beinmodells um 1 cm zunimmt. Diese Umfangszunahme des Beines, welche sich an der Definition der Stiffness orientiert, kann herangezogen werden, um durch die erhobenen Druckmessungen die einzelnen Stiffness-Indizes zu berechnen. Validitätsprüfungen konnten nachweisen, dass die erhobenen Daten mit den unter Laborbedingungen angewandten Hohenstein-Messungen korrelieren [15].

## Allgemeingültige technische Empfehlungen zur Durchführung von Druckmessungen

Unabhängig von den verschiedenen Messmethoden wurde in einem Konsensus-Statement [18] eine Reihe von Empfehlungen zur Durchführung von Druckmessungen unter Kompressionsmitteln festgehalten (vgl. Tab. 2), sowie die Eigenschaften des optimalen Messsensors definiert (vgl. Tab. 3).

## Stiffness im Kontext verschiedener Kompressionsmittel

Die medizinische Kompressionstherapie bietet eine Reihe an verschiedenen Kompressionsmitteln. Hierunter fallen medizinische Kompressionsstrümpfe, Kurzzugbinden, Langzugbinden, Multikomponentensystem und adjustierbare Kompressionssysteme [1–4, 8, 18, 22, 23, 29].

Diese können aufgrund unterschiedlicher Materialeigenschaften, auch innerhalb einer Kompressionsklasse und Produktgruppe, verschiedene Level an Stiffness aufweisen und somit unter dynamischen Aspekten unterschiedlich effektiv hinsichtlich der Ausübung eines Massageeffekts der unteren Extremität sein [3, 10–12].

### Medizinische Kompressionsstrümpfe

Innerhalb der Gruppe der medizinischen Kompressionsstrümpfe findet sich eine Vielzahl von Produkten unterschiedlicher Eigenschaften. Produktdefinierend sind hierbei die Kompressionsklasse, Strumpflänge, Herstellungsart oder Materialfestigkeit [29].

Die Kompressionsklassen werden in Deutschland nach der RAL-Norm (RAL-GZ 387) unterteilt und gliedern sich in die Klassen I–III in der Versorgung mit Rundstrickstrümpfen und in die Klassen I–IV in der Versorgung mit Flachstrickstrümpfen [26].

In der Produktion werden heutzutage v. a. synthetische Garne aus Polyurethan (Elastan) verwendet, welche den elastischen Kern von Schussfaden und Maschenfaden bilden. Ummantelt werden sie von Polyamidgarnen, welche die Widerstandsfähigkeit der Strümpfe und das Tragegefühl auf der Haut definieren [29].

Die Schussfäden bedingen aufgrund ihrer Stärke die Kompression eines Strumpfes, während die Maschenfäden v. a. für das subjektive Empfinden beim Tragen verantwortlich sind [29].

Rundstrickkompressionsstrümpfe zeichnen sich in ihrer Herstellung durch kontinuierliche, unter Spannung stehende [11] Schussfäden aus, welche spiralförmig durch das Material ziehen und von Maschenfäden in einer konstanten Dichte von 20 Maschen/Zoll zusammengehalten werden. Sie sind in der Regel dünn im Material und sehr elastisch (SSI < 10 mm Hg) [26].

Flachstrickstrümpfe hingegen verwenden Schussfäden, welche parallel zueinander verlaufen und über eine zentrale Naht entlang des dorsalseitigen Kompressionsstrumpfes zusammengehalten werden. Die Passform der Flachstrickstrümpfe wird über eine variable Anzahl an Maschen gesteuert, welche in der Regel mit 14 Maschen/Zoll eine geringere Dichte aufweisen als bei Rundstrickstrümpfen, was zur Reduktion der transversalen Elastizität führt [26]. Dies bewirkt in Kombination mit den dickeren Garnen der Flachstrickstrümpfe eine höhere Stiffness des Materials im Vergleich zu rundgestrickten Kompressionsstrümpfen [26]. Zudem bieten Flachstrickstrümpfe über ihre variable Anzahl an Maschen die Möglichkeit einer individuellen Anpassung, wodurch Extremitäten mit großen Umfangsdifferenzen versorgt werden können [26].

### Kompressionsbandagen

Kompressionsbandagen werden in Deutschland ebenfalls über die RAL-GZ 387 normiert [29].

Neben der Spannung, Stärke und Elastizität spielt v. a. die Dehnbarkeit eine entscheidende Rolle in der Produktdefinition [29]. Ein weiteres Akronym zur Beschreibung der Eigenschaften von Kompressionsbandagen bildet das von Partsch et al. geprägte „P-LA-C-E“, mit dem Fokus auf Druck („pressure“), Überlappung („layers“), Komponenten („components“) und Elastizität („elastic“) von Kompressionsbinden [27]. Die Dehnbarkeit bzw. Elastizität stellt hierbei die entscheidende Größe zur Einteilung in die 2 Gruppen der elastischen (Langzugbinden) und unelastischen (Kurzzugbinden) Kompressionsbandagen dar. Langzugbinden zeichnen sich über eine potenzielle Dehnbarkeit des Materials von über 100 % aus, während Kurzzugbinden eine maximale Dehnung von weniger als 100 % zulassen [14, 29].

Maßgeblich hierfür sind die in der Produktion verwendeten Materialien. So bestehen Langzugbinden zumeist aus synthetisch-elastischen Materialien wie Gummi, Lycra oder Elastan [24], während Kurzzugbinden in der Regel aus unelastischen, stark verzwirbelten Leinen- oder Baumwollgarnen gefertigt werden [24, 29].

In der klinischen Praxis führt dies zu einer höheren Stiffness (SSI > 10 mm Hg) von Kurzzugbinden im Vergleich zu Langzugbandagen (SSI < 10 mm Hg) [22, 24], was Einfluss auf ihre therapeutischen Indikationen, Vorteile und Limitationen hat.

Kurzzugbinden zeichnen sich im klinischen Alltag v. a. durch einen hohen Arbeitsdruck aus, was bei mobilen Patienten durch die oszillierenden Druckspitzen unter Bewegung zum Massageeffekt an der unteren Extremität und damit zur Entstauung des Gewebes führt [22]. Sie verbessern signifikant effektiver als Langzugbinden das venöse Ejektionsvolumen sowie die venöse Ejektionsfraktion [16]. Gleichzeitig weisen Kurzzugbinden einen niedrigen Ruhedruck aus, wodurch sie tags wie auch nachts besser von Patienten toleriert werden und sich bis zu einem gewissen Punkt besonders auch in der Therapie von Patienten eignen, welche neben lymphatisch-venösen Erkrankungen an einer peripheren arteriellen Verschlusskrankheit leiden [23, 24].

Zu berücksichtigen in der Handhabung von Kurzzugbinden ist v. a. die schnelle Entstauung und die damit einhergehende rasche Abnahme des Beinumfangs. Dies führt mit der Zeit zu einer Reduktion des Kompressionsdrucks sowie oftmals zum Verrutschen der Bandage mit der Notwendigkeit von Neuanlagen im Tagesverlauf [23, 29].

Langzugbinden zeichnen sich im Gegensatz hierzu durch einen relativ geringen Arbeitsdruck bei hohem Ruhedruck aus, wodurch sie eine weniger effektive Entstauung der unteren Extremität bewirken [24]. Insbesondere im Management vom Lymphödemen sind sie deshalb weniger geeignet [24] und erreichen in der Regel keine Normalisierung der venösen Pumpfunktion bei chronisch venöser Insuffizienz [16]. Versuche, die hämodynamischen Auswirkungen von Langzugbandagen über eine höhere Spannung bei der Anlage zu verbessern, brachten keine Effekte, förderten jedoch Unbehagen bei den Patienten [16]. Auch über Nacht berichten Patienten, bedingt durch den hohen Ruhedruck von Langzugbinden, vermehrt von unangenehmen bis schmerzhaften Missempfindungen [16, 24]. Im Kontrast hierzu steht die Eigenschaft von Langzugbandagen, über Zeiträume von bis zu einer Woche am Patientenbein verbleiben zu können, ohne dass ihr Sitz maßgeblich beeinträchtigt wird [24, 29], da es durch die verzögerte Entstauung sowie durch die elastischen Eigenschaften des Materials zur Anpassung an die Unterschenkelumfangsänderungen kommt [24, 29].

### Mehrkomponentensysteme

Hierunter fallen Kompressionsmittel, welche aus multiplen Teilkomponenten bestehen. Diese können sowohl aus mehreren voneinander getrennten Lagen eines Produktsystems bestehen (z. B. UrgoK2) [25], aus mehreren Lagen verschiedener Produkte (z. B. Kombinationen zwischen Kompressionsstrümpfen und Kompressionsbinden) [1, 5, 22, 23] oder aus in einer einzelnen Lage vereinten Teilkomponenten (z. B. UrgoK1) [25].

Mehrkomponentensysteme sind v. a. in der Behandlung von chronischen Ulzera eine Standardmodalität und dienen in der Entstauungsphase der unteren Extremität als Mittel der Wahl [1, 22, 23, 25, 26, 28]. Hierbei haben Systeme mit hoher Stiffness einen größeren therapeutischen Effekt gezeigt als solche mit geringer Stiffness [1, 16, 22, 23, 27]. Über den modularen Aufbau kommt es zwischen den einzelnen Lagen von Mehrkomponentensystem zu Reibungskräften [19]. Dieser Einflussfaktor erhöht insbesondere die dynamische Stiffness [19] und trägt dazu bei, dass initial elastische Einzelkomponenten in ihrer kombinierten Anwendung die Produkteigenschaften von unelastischen Materialien annehmen [27].

Zu den am häufigsten angewandten Mehrkomponentensystemen zählen Kompressionssysteme aus 2 unterschiedlichen Bandagen, in der Regel bestehend aus einer Polsterschicht (z. B. Watte) und einer Kompressionsschicht. Hierbei sind eine oder beide Lagen selbsthaftend (kohäsiv) und führen damit zum geringeren Verrutschen und damit länger aufrechterhaltener konstanter Kompression [23, 29]. Mehrkomponentensysteme bieten hierdurch gegenüber Kompressionsverbänden nur aus Kurzzugbinden den Vorteil eines geringeren Druckverlustes über die Zeit bei insgesamt vergleichbaren statischen Stiffness-Indizes [23].

Eine weitere Form der Mehrkomponentenkompression stellt die kombinierte Anwendung verschiedener Kompressionsstrümpfe und Kompressionsbinden übereinander dar.

Hierbei erzielen Kombinationen aus 2 Kompressionsstrümpfen, die Kombination aus Kompressionstrumpf und Kompressionsbinde sowie die Kombination aus 2 Kompressionsbinden einen signifikanten Anstieg des Kompressionsdrucks und der Stiffness [22]. Kurzzugmaterialien führen als zweite Lage sowohl bei Kompressionsstrümpfen als auch bei Kompressionsbinden zu einem merklicheren Anstieg der Stiffness [22], während Langzugbinden als äußere Schicht zu einer Reduktion der Stiffness führen [22]. In einem weiteren Ansatz konnte die Kombination aus Kompressionsstrumpf mit darüber modular anwendbaren Leggins einen Anstieg von Kompressionsdruck und dynamischem Stiffness-Index zeigen [1].

### Adaptive Kompressionssysteme

Adaptive Kompressionssysteme existieren in einer Vielzahl von verschiedenen Ausführungen, so z. B. in Form von sich überlappend angelegten Kompressionssegmenten, welche über ein gemeinsames Verbindungsstück zusammengehalten werden (z. B. ReadyWrap (Lohmann & Rauscher GmbH & Co. KG, Neuwied, Deutschland), circaid juxtafit (medi GmbH & Co. KG, Bayreuth, Deutschland), Juzo Compression Wrap 6000 (Julius Zorn GmbH, Aichach, Deutschland)) [8], oder nach gleichem Prinzip angelegte Kompressionssysteme, welche zusätzlich eine Inflation von Luft in die einzelnen Segmente erlauben [2]. All diese Systeme zeichnet eine einfachere Handhabung beim Anlegen aus, wodurch sie von Patienten selbst angebracht und nachjustiert werden können, was wiederum einen positiven Einfluss auf die Compliance und Therapieadhärenz nimmt [8]. Dies ist ein Vorteil gegenüber medizinischen Kompressionsbandagen, bei denen der therapeutische Effekt maßgeblich von der korrekten, mitunter fehleranfälligen Anlage abhängt [2, 30] und ein Vorteil gegenüber medizinischen Kompressionsstrümpfen, bei denen eine patienteneigene Adjustierbarkeit nicht gegeben ist. In Bezug auf Kompressionsdruck und Stiffness zeigten sich in der Untersuchung eines adaptiven Kompressionswickelsystems mit integrierten und druckadaptiven Luftpolstern vergleichbare Stiffness-Indizes wie in der Anwendung von unelastischen Kompressionsbandagen [2].

Adaptive Kompressionssysteme können vor diesem Hintergrund mit einer vergleichbaren Effektivität hinsichtlich der Verbesserung hämodynamischer Parameter eingesetzt werden wie unelastische Kompressionsbandagen [2] und erzielen insbesondere in der Entstauungstherapie der unteren Extremität gute Effekte [8].

## Stellenwert der Kompressionstherapie aus Patientensicht

Kompressionsmittel stellen eine Standardmodalität in der konservativen Behandlung venös-lymphologischer Erkrankungen dar [1–4, 26, 29].

Um ein Abbild der Patientenmeinungen zur Therapie mit medizinischen Kompressionsstrümpfen zu erhalten, erfolgte 2023 eine repräsentative Umfrage unter Befragung von insgesamt 1300 Teilnehmer*innen in Deutschland, welche medizinisch verordnete Hilfsmittel im Alltag nutzen [31].

Es zeigte sich unter den hochgerechnet ca. 5,9 Mio. Anwendern von Kompressionsstrümpfen in Deutschland ein überwiegend positiver Ausblick auf die Durchführung einer Kompressionstherapie [31]; 81 % der Befragten gaben an, ihre Strümpfe nahezu täglich zu tragen und dies im Durchschnitt seit bereits 6,9 Jahre; 87 % gaben an, zufrieden oder sehr zufrieden mit ihren Kompressionsstrümpfen zu sein, wobei 88 % viel bzw. sehr viel Nutzen aus der Therapie zogen. So gaben 65 % der Befragten an, einen Rückgang von Schwellungen bemerkt zu haben, 48 % berichteten von einer Reduktion von Schmerzen, 44 % von einer Reduktion schwerer oder müder Beine und 40 % von einem reduzierten Spannungsgefühl der unteren Extremitäten. Ebenfalls eine hohe Prozentzahl der Befragten (83 %) zeigte sich zufrieden mit der Passform der Strümpfe. Hinsichtlich der täglichen Handhabung betonten 49 %, ihre Kompressionsstrümpfe problemlos an- und ausziehen zu können [31].

Entgegen diesen insgesamt positiven Aussichten finden sich in der Literatur Angaben von 40 % der Kompressionsmittelträger, welche ihre Therapie nicht regelmäßig durchführen können oder wollen [32]. Als häufigste Ursachen werden Schwierigkeiten beim Anziehen, ein Gefühl der Einengung sowie ein Austrocknen der Haut [32] genannt. Weiterhin spielen ästhetische Aspekte wie auch durch zu starke Kompression entstehendes Unbehagen oder Schmerzen eine Rolle bei der Therapieadhärenz [11, 15, 16].

## Stiffness im Alltag der Kompressionstherapie

Ein Kernaspekt in der Effektivität der medizinischen Kompressionstherapie stellt die regelmäßige Anwendung dar. Diese wird erreicht durch ein Gleichgewicht aus notwendiger Kompression und Tolerierbarkeit der Therapie seitens der Patienten [27], weshalb die Internationale Gesellschaft für Lymphologie den höchsten vom Patienten tolerierbaren Kompressionsdruck als Zielwert empfiehlt [12, 30], während die deutsche Leitlinie zur medizinischen Kompressionstherapie [33] sowie die deutsche Leitlinie zum Lipödem [34] den niedrigsten wirksamen Kompressionsdruck empfehlen [33, 34]. Die Stiffness als zusätzlicher Parameter von Kompressionsmaterialien stellt eine Einflussgröße dar, welche unabhängig vom Kompressionsdruck einen Einfluss auf die Therapieeffektivität bei Patienten nimmt, da insgesamt eine Korrelation zwischen der Höhe der Stiffness und der Ejektionsfraktion der Muskel-Venen-Pumpe besteht [7]. Die optimalen Werte für Kompressionsdrücke und Stiffness in der klinischen Praxis für die verschiedenen Indikationen wurden bis dato jedoch nicht abschließend definiert [15, 26].

Bei habituellen und leichtgradigen venösen Ödemen scheinen Kompressionsdrücke von 20 mm Hg ausreichend [2, 5], während beim Vorliegen von trophischen Hautveränderungen oder venösen Ulzera Kompressionsdrücke von > 40 mm Hg angezeigt seien [2]. Insbesondere chronische Gewebsschädigungen wie das Ulcus cruris venosum profitieren aufgrund der Korrelation zwischen hoher Stiffness und damit einhergehend hoher Ejektionsfraktion der Venen [7] von einer hämodynamischen Verbesserung der Mikrozirkulation im Kapillarstromgebiet, wodurch ihr Auftreten reduziert und ihre Abheilung gefördert wird [2–4]. Bei Vorliegen einer chronischen venösen Insuffizienz dienen Ruhedrücke im Liegen von 40 mm Hg am B1-Punkt als Zielwert, wobei Drücke von 50–70 mm Hg bei Verwendung von Kompressionsbandagen als Idealbereich beschrieben werden [5, 23, 27].

Hinsichtlich der Stiffness ist die Effektivität einer Kompressionstherapie umso effektiver je höher die Stiffness der angewandten Materialien [4, 6, 9–13, 15, 16, 18, 20–23] und je mobiler die Patienten sind, da beide Faktoren den unter dynamischen Situationen auftretenden Massageeffekt der unteren Extremität bedingen [10, 12–16, 18–22].

In der täglichen Anwendung von Kompressionsmitteln würde dies die Möglichkeit bieten, mobile Patienten mit Produkten einer geringeren Kompressionsklasse und hoher Stiffness auszustatten, um bei erhaltener Patientencompliance einen adäquaten klinischen Therapieeffekt zu erzielen [12].

Zu bedenken bleiben in diesem Kontext die unterschiedlichen Level an Stiffness nicht nur zwischen den einzelnen Produkten einer Kompressionsgruppe und -klasse [10, 12, 20], sondern auch zwischen den verschiedenen Arten einer Kompressionstherapie.

Unelastische Kompressionsmaterialien wie Kurzugbinden zeigen hohe Stiffness-Werte und eine adäquate therapeutische Effektivität [9, 13–16, 20–24, 28]. Langzugbinden weisen aufgrund ihrer elastischeren Materialeigenschaften geringere Stiffness-Werte auf, weshalb sie in der Therapie der chronisch venösen Insuffizienz und Lymphödembehandlung weniger geeignet sind [1, 2, 5, 7, 13, 16, 24]. Innerhalb der Gruppe der Kompressionsstrümpfe zeigten solche mit Flachstrickherstellung eine höhere Stiffness als rundgestrickte Kompressionsstrümpfe [12, 20, 26].

Dennoch zeigt sich in der Produktkategorie der Rundstrickkompressionsstrümpfe eine breite Variabilität an Elastizität, sodass Strümpfe mit eher elastischen Eigenschaften als auch solche mit höherer Stiffness vermarktet werden [3, 12]. Insbesondere die rundgestrickten MKS mit höheren Stiffness-Indizes eignen sich in der Kompressionstherapie phlebologischer Indikationen und werden derzeit seitens der Hersteller oft als Produkte mit starkem Rundstrick vermarktet. Weitere Modalitäten wie Multikomponentensysteme bieten eine Vielzahl an individuellen Möglichkeiten in der Patientenversorgung. Kombinationen aus verschiedenen Produkten wie Kompressionsstrümpfen, Kurzzugbinden, Langzugbinden oder Leggins bieten den Vorteil einer hohen Stiffness und Druckamplitude [1, 22, 23] bei gleichzeitiger Möglichkeit zur patienteneigenen, individuellen Justierung [1, 22].

So kann insbesondere bei Patienten mit Schwierigkeiten im Anziehen von Kompressionsstrümpfen einer hohen Kompressionsklasse auf Mehrkomponentensysteme ausgewichen werden, ohne einen Verlust der Therapieeffektivität in Kauf zu nehmen [22].

## Fazit

- Die Stiffness stellt einen gesonderten Prädikator für die Effektivität einer Kompressionstherapie dar und kann im klinischen Alltag gezielt eingesetzt werden, um die optimale Kompressionsmittelversorgung für die unterschiedlichen Indikationen und Patienten auszuwählen.
- Aufgrund der hohen Korrelation zwischen dem statischen und dynamischen Stiffness-Index sowie der einfacheren Bestimmung des statischen Stiffness-Index ist dessen Angabe im alltäglichen Gebrauch als ausreichend anzusehen.
- Wünschenswert wären in Zukunft ein Konsensus zur Ermittlung der Stiffness von medizinischer Kompressionsversorgung. Dies würde es den Herstellern von Kompressionsmitteln ermöglichen, Stiffness als Produktinformation zu etablieren, um die aktuell bestehende Informationslücke auf dem Markt zu schließen und es Medizinern zu erleichtern, die bestmögliche Versorgung der einzelnen Patienten zu gewährleisten. Bis dahin empfehlen sich weitere Arbeiten zur Bestimmung der Stiffness von aktuell auf dem Markt erhältlicher Kompressionsware.

### Kernaussagen.

- Die Stiffness stellt neben dem Kompressionsdruck eine entscheidende Produkteigenschaft von Kompressionsmaterialien dar.
- Stiffness beschreibt die elastischen Eigenschaften eines Materials und ist definierend für das Verhalten und die Effektivität einer Kompressionstherapie unter dynamischen Bedingungen.
- Produkte der gleichen Kompressionsgruppe und Kompressionsklasse können unterschiedliche Level an Stiffness aufweisen.
- Je höher die Stiffness eines Materials desto größer sind die positiven hämodynamischen Effekte auf die untere Extremität.
- Der statische Stiffness-Index (SSI) berechnet sich als Differenz aus dem Anpressdruck im Stand und dem Ruhedruck im Liegen und stellt eine einfach bestimmbare Messgröße in vivo dar.
